# A non-randomised pilot study of the Solutions for Medication Adherence Problems (S-MAP) intervention in community pharmacies to support older adults adhere to multiple medications

**DOI:** 10.1186/s40814-020-00762-3

**Published:** 2021-01-07

**Authors:** D. E. Patton, C. J. Pearce, M. Cartwright, F. Smith, C. A. Cadogan, C. Ryan, E. Clark, J. J. Francis, C. M. Hughes

**Affiliations:** 1 School of Pharmacy, Queen’s University Belfast, Belfast, UK; 2 School of Health Sciences, City University of London, London, UK; 3 School of Pharmacy, University College London, London, UK; 4 School of Pharmacy and Biomolecular Sciences, Royal College of Surgeons in Ireland, Dublin, Ireland; 5 School of Pharmacy & Pharmaceutical Sciences, Trinity College Dublin, Dublin, Ireland

**Keywords:** Medication adherence, Polypharmacy, Theory, Behaviour change, Community pharmacy, Pilot study, Technology, Complex intervention

## Abstract

**Background:**

Older patients prescribed multiple medications commonly experience difficulties with adherence. High-quality evidence on interventions targeting older patients is lacking. Theory is rarely used to tailor adherence solutions. This study aimed to pilot test a novel intervention, developed using the Theoretical Domains Framework, which guides community pharmacists in identifying adherence barriers and delivering tailored solutions (behaviour change techniques). Key study procedures (e.g. recruitment, data collection) for a future randomised controlled trial (cRCT) were also assessed.

**Methods:**

Using purposive sampling, this non-randomised pilot study aimed to recruit 12 community pharmacies (six in Northern Ireland; six in London, England). Pharmacists were trained to deliver the intervention to non-adherent older patients (maximum 10 per pharmacy; target *n* = 60-120) aged ≥ 65 years (reduced to 50 years due to recruitment challenges) and prescribed ≥ 4 regular medicines. The intervention, guided by an iPad web-application, was delivered over 3-4 face-to-face or telephone sessions, tailored to specific barriers to adherence. We assessed the feasibility of collecting adherence data (primary outcome: self-report and dispensing records), health-related quality of life (HRQOL) and unplanned hospitalisations (secondary outcomes) at baseline and 6-months. The final decision on progressing to a cRCT, using pre-defined ‘stop-amend-go’ criteria, is presented.

**Results:**

Fifteen pharmacists from 12 pharmacies were recruited and trained. One pharmacy subsequently dropped out. Sixty patients were recruited (meeting the ‘Amend’ progression criteria), with 56 receiving the intervention. Adherence barriers were identified for 55 patients (98%) and a wide range of behaviour change solutions delivered (median: 5 per patient). Self-report and dispensing adherence data were available for 37 (61.7%) and 44 (73.3%) patients, respectively. HRQOL data were available for 35 (58.3%) patients. GP-reported and self-reported hospitalisations data were available for 47 (78.3%) and 23 (38.3%) patients, respectively. All progression concepts were met (nine ‘Go’ and three ‘Amend’ criteria).

**Conclusion:**

This study demonstrates the feasibility of key study procedures (e.g. pharmacy recruitment) and delivery of a tailored adherence intervention in community pharmacies. However, modifications are required to enhance issues identified with patient recruitment, retention and missing data. A future definitive cRCT will explore the effectiveness of the intervention.

**Trial registration:**

ISRCTN, ISRCTN73831533, Registered 12 January 2018.

**Supplementary Information:**

The online version contains supplementary material available at 10.1186/s40814-020-00762-3.

## Key messages regarding feasibility

1. What uncertainties existed regarding feasibility?

- Uncertainties remained regarding the feasibility of pharmacy sampling/recruitment, patient screening/recruitment, intervention delivery and outcome data collection procedures.

2) What are the key feasibility findings?

- This study has demonstrated the feasibility of pharmacy sampling/recruitment procedures and intervention delivery in the community pharmacy setting. Challenges were identified with patient recruitment procedures, patient retention and missing data.

3) What are the implications of the feasibility findings for design of the main trial?

- The findings from this study will inform changes required to study procedures in the main trial including modifications to reduce the amount of missing data and enhance patient recruitment/retention (e.g. support from a clinical research network)

## Background

Medication adherence, defined as taking medications in accordance with the prescribers’ directions, can be problematic, particularly in older patients (≥ 65 years) prescribed multiple medicines to treat a range of long-term conditions [1]. A recent study in Ireland found that 31% of older people living with multimorbidity (≥ 2 chronic conditions) were non-adherent, with non-adherence rates varying across conditions and treatments [2]. Polypharmacy, commonly defined as the prescribing of four or more medications, is associated with low adherence [3]. Medication non-adherence has a negative impact on both individual patients and the wider healthcare system as it can result in inadequate disease control, decreased quality of life, increased morbidity, hospitalisations and increased healthcare costs [1, 4]. For example, a 2012 report from the Institute for Healthcare Informatics estimated that total costs from non-adherence amounted to approximately US$270 billion per year globally [5].

To date, a plethora of interventions have been developed to address the challenge of medication non-adherence in adults, but these have shown only limited effectiveness in improving adherence and clinical outcomes [6]. A recent Cochrane review of adherence interventions, designed to target older patients prescribed multiple medications, found a lack of high-quality evidence on intervention effectiveness and interventions were not commonly tailored to individual patient-reported barriers to adherence [1]. It has been proposed that psychological theories may guide the development of more effective complex adherence interventions by targeting causal determinants of behaviour [7]. However, a systematic review of interventions targeting older patients prescribed multiple medications found that theory was rarely used to guide the intervention content [8].

The S-MAP (Solutions for Medication Adherence Problems) intervention is a theory-based intervention that has been systematically developed in line with the United Kingdom (UK) Medical Research Council’s (MRC) guidance for complex interventions [9]. The intervention is tailored to each individual patient’s underlying reasons for non-adherence. Using the Theoretical Domains Framework [10], previous research involving focus groups with older patients identified eight key domains linked to non-adherence (beliefs about consequences; motivation and goals; environmental context and resources; knowledge; memory, attention and decision processes; social influences; behavioural regulation; nature of the behaviours) [11]. These domains were then mapped to 11 behaviour change techniques (BCTS) using established mapping resources [12, 13] (e.g. information about health consequences; prompts/cues; goal-setting—behaviour). BCTs are ‘…the smallest components of behaviour change interventions that on their own in favourable circumstances can bring about change’ [14]. The 11 BCTs identified using this systematic approach were then developed into an intervention package for delivery by community pharmacists to older patients prescribed multiple medications [15], and a preliminary feasibility study was conducted in two community pharmacies in Northern Ireland (NI) (https://doi. org/10.1186/ISRCTN17966504) [16]. Although the intervention showed potential, this study identified the need for modification of the intervention and refining of study procedures. For example, the original intervention included a paper-based adherence assessment tool to identify adherence barriers and solutions (i.e. BCTs). However, feedback from pharmacists indicated that large amounts of paperwork would not be acceptable; hence, an electronic version of the adherence assessment tool warranted development and testing.

The current paper reports a multi-centre non-randomised pilot study of an enhanced intervention (S-MAP) conducted in community pharmacies in NI and London, England. This pilot study, which contained an intervention group only (with no control group), was designed to examine important study procedures (e.g. patient recruitment) on a larger scale, in advance of a larger cluster randomised controlled trial (cRCT) of the S-MAP intervention.

The main objectives of the current study were as follows:

1. Test approaches to sampling, recruitment and retention of community pharmacies
2. Test approaches to screening, recruitment and retention of patients in community pharmacies
3. Assess the delivery of the S-MAP intervention (guided by an iPad web-application) in community pharmacies in NI and London
4. Explore the feasibility of collecting data for outcome assessment

The final decision on whether to proceed to a definitive trial of effectiveness (cRCT), based on the pre-defined progression (Stop-Amend-Go) criteria originally outlined in the study protocol [17], is also presented.

## Methods

### Study design and setting

A non-randomised multi-centre pilot study (intervention only group, i.e. no control) was conducted in the community pharmacy setting in NI and London.

### Sampling and recruitment of community pharmacies

The study aimed to recruit 12 community pharmacies across NI and London with a view to 10 pharmacies completing the study (i.e. assuming a retention rate of 80%). Using maximum variation sampling, the study aimed to recruit six pharmacies in NI, with at least one from each of the five Health and Social Care Trust areas (HSCTs; the administrative areas for healthcare provision in NI). In London, six Clinical Commissioning Groups (CCGs) were purposively selected from 32 in the region with the aim of recruiting one pharmacy from each. CCGs from both inner and outer areas of London were selected taking into consideration the level of deprivation [i.e. Index of Multiple Deprivation (IMD) CCG rank scores whereby a rank of 1 indicates the most deprived CCG area, and a rank of 191 indicates the least deprived CCG area] [18]. The selected CCGs (and ranks) were Newham (11th), Lambeth (38th), Camden (94th), Bromley (162nd), Barnet (138th) and Richmond (186th) [18]. The 2019 England IMD decile [18] (most deprived area = score of 1, least deprived area = score of 10) and 2017 NI Multiple Deprivation Measure [19] (MDM; most deprived area = score of 1 and least deprived area = score of 890) score for the location (i.e. postcode) of each recruited community pharmacy in London and NI, respectively, are presented in the “Results” section.

The study also aimed to include pharmacies in urban, suburban and rural areas and different types/sizes of pharmacies (independently-owned, small and large chains). Region-specific definitions were used to categorise the type of pharmacy as NI has relatively few chains with more than 100 pharmacies, compared to England (see Additional file 1 for definitions) [20, 21].

Letters seeking expressions of interest were mailed to 60 pharmacies in each region (12 per HSCT area in NI and 10 per CCG in London). Pharmacies in the mailing list were strategically selected to include a broad range of pharmacy types, social deprivation levels and different types of area (e.g. rural/urban). Members of the research team (DP, EC) contacted pharmacists who returned reply slips to discuss participation in the study. Those who did not return reply slips were contacted via telephone to enquire about participation. Non-responding pharmacies were purposively selected and contacted to provide maximum variation within the sample (e.g. chains and independently-owned pharmacies). Community pharmacies were eligible to participate if they had a suitable consultation area (e.g. private area with seating), access to Wi-Fi and printing facilities. Pharmacists were eligible to participate if they worked on a regular basis in the pharmacy (e.g. > 2 days per week). Pharmacy support staff were also eligible for participation, to support pharmacists with study procedures (e.g. patient recruitment). Interested pharmacies were emailed/posted a study information sheet and consent form and meetings were arranged to discuss participation and obtain written informed consent. Pharmacists were invited to attend a 1-day training workshop to equip them to deliver the study. An online training package was developed, using the Moodle® platform, which included a version for pharmacists who were unable to attend the workshop and a version for pharmacy support staff. Pharmacies were provided an honorarium of £500(NI)/£600(London) for participation in the study and each pharmacist was awarded a certificate for their Continuing Professional Development portfolio. Pharmacies were also provided an additional £30 for each patient they recruited into the study and delivered the intervention to (up to an additional £300 per pharmacy).

### Screening and recruitment of patients

Patients were eligible for participation in the study if they were (1) 65 years or older (amended during the study to 50 years, see below), (2) prescribed four or more regular medications (polypharmacy), excluding ‘when required’ medicines or those with variable dosing directions (e.g. take once or twice daily), (3) receiving prescriptions from the recruited pharmacy for at least 12 months, (4) identified as non-adherent (see below), (5) living in their own home, (6) able to provide written informed consent and (7) receiving all regular medications from the recruited pharmacy. Patients were excluded if they were prescribed medications for the management of dementia as the intervention was not designed to address the additional challenges faced by this patient group.

Initially, a two-stage screening process was trialled. In stage 1, pharmacists screened Patient Medication Records (PMR) or prescriptions to identify patients who met eligibility criteria 1-3. Patients prescribed medications for dementia were excluded at this stage. In stage 2, patients who met criteria 1-3 were approached either in the pharmacy or mailed a questionnaire to identify if they were non-adherent (criterion 4), using two self-report measures—Medication Adherence Report Scale (MARS)-5 [22] and a single item adapted from Lu et al. [23]. Total scores for MARS-5 (‘Always’ = 1; ‘Often’ = 2; ‘Sometimes’ = 3; ‘Rarely’ = 4; ‘Never’ = 5) range from 5 to 25 with higher scores denoting higher adherence. The Lu item was scored from 0 to 100% (‘Very poor’ = 0%; ‘Poor’ = 20%; ‘Fair’ = 40%; ‘Good’ = 60%, ‘Very good’ = 80%, ‘Excellent’ = 100%) [23]. Patients who scored < 80% on the Lu item and/or < 25 on MARS-5 were deemed non-adherent. The cut-off point for the Lu item is in line with the 80% cut-off commonly used in adherence research [24, 25]. The cut-off point of < 25 for MARS-5 has been used previously in the literature [26, 27]. The pharmacist also confirmed that the patient met criteria 5-7 in stage 2. Eligible patients were invited to take part and written informed consent was obtained by the pharmacist or trained support staff member prior to session 1 (see ‘Intervention overview’ section).

Due to challenges with patient recruitment observed during the first 4 months of the study, the screening approach was modified. Based on pharmacist feedback, patients did not always report non-adherence on the questionnaire despite dispensing records highlighting potential problems. Following an approved amendment to the study protocol, the self-report adherence questionnaire was removed from the eligibility screening process. The questionnaire was, however, still administered as part of the baseline outcome assessment following recruitment (see ‘Outcome data collection’ section). In the modified screening approach, an assessment of the patient’s adherence was made based on informal discussions between the patient and pharmacist and/or information obtained from dispensing records (e.g. medication supply gaps). Information flyers were also distributed in the pharmacy and posters displayed to advertise the study and enhance recruitment rates. Additionally, as suggested by the pharmacists, to improve recruitment rates the age limit for participation was lowered from 65 to 50 years. Initially, a period of 6 months was assigned for patient recruitment, however, due to the challenges experienced this was extended to 12 months to allow for an assessment of the modifications made. Collection of outcome data was originally planned to take place at baseline, 6 months and 12 months from baseline, but due to the extended recruitment period, the 12-month follow-up time point was removed.

### Sample size

As this was a pilot study that did not aim to assess intervention effectiveness against statistical criteria, a power calculation was not conducted. Instead, in anticipation of a future cluster RCT, a pragmatic target of 10 patients per community pharmacy (i.e. a maximum of 120 patients in total) was used for this study. A minimum of 60 patients was deemed sufficient to meet the ‘Amend’ progression criterion for patient recruitment (see ‘Progression criteria’). Based on the experience of the research team, this sample size coupled with the sampling approach outlined above would provide sufficient data to meet the objectives of this pilot study.

### Retention of patient participants

Retention rates were determined based on the proportion of retained participants who were assessed with valid primary outcome data [28]. Patients were considered retained in the study if they had data suitable for analysis for the primary outcome of medication adherence (see ‘Outcome data collection’ section).

### Intervention overview

The S-MAP intervention is an individually tailored medication adherence intervention that was designed for delivery by community pharmacists and guided by a web-application (hereafter referred to as an ‘app’), which contained an adherence assessment tool. Each pharmacist was provided with individual log-in details and an iPad to access the app during the study (to allow sessions to be conducted in consultation rooms/areas without computer equipment). Each recruited patient was entered into the system using a unique study identification number. The patient’s medications were manually recorded on the app in advance of their first session and the medication history confirmed with the patient at Session 1 and their general practitioner (GP) if required (e.g. to confirm the correct dosage).

### Intervention content (behaviour change techniques)

As reported in the protocol paper [17], the intervention content (BCTs) in the app was coded and compared to the intervention content in the previous paper-based version. The purpose was to ensure all anticipated BCTs were present in the app-supported version of the intervention and to identify any additional, unanticipated BCTs. This BCT coding exercise was undertaken by members of the research team (SC, CC, JF, EC) who were not directly involved in the original content development. Four additional BCTs were identified during this process and were added to the intervention specification, bringing the total number of BCTs to 15. The final version of the S-MAP app tested in the current study was also coded for BCTs and one further BCT was identified (the ‘Instruction on how to perform the behaviour’ section) [29]. This brings the total number of BCTs in the app to 16 (see Additional file 2): (1) problem solving, (2) self-monitoring (behaviour), (3) feedback on behaviour, (4) social support (unspecified), (5) social reward, (6) goal-setting-behaviour, (7) action planning, (8) review of behaviour goal, (9) social support (practical), (10) goal-setting (outcome), (11) review of outcome goal, (12) information about health consequences, (13) prompts/cues, (14) restructuring the physical environment, (15) adding objects to the environment and (16) instruction on how to perform the behaviour.

### Session overview

**Fig. 1:**
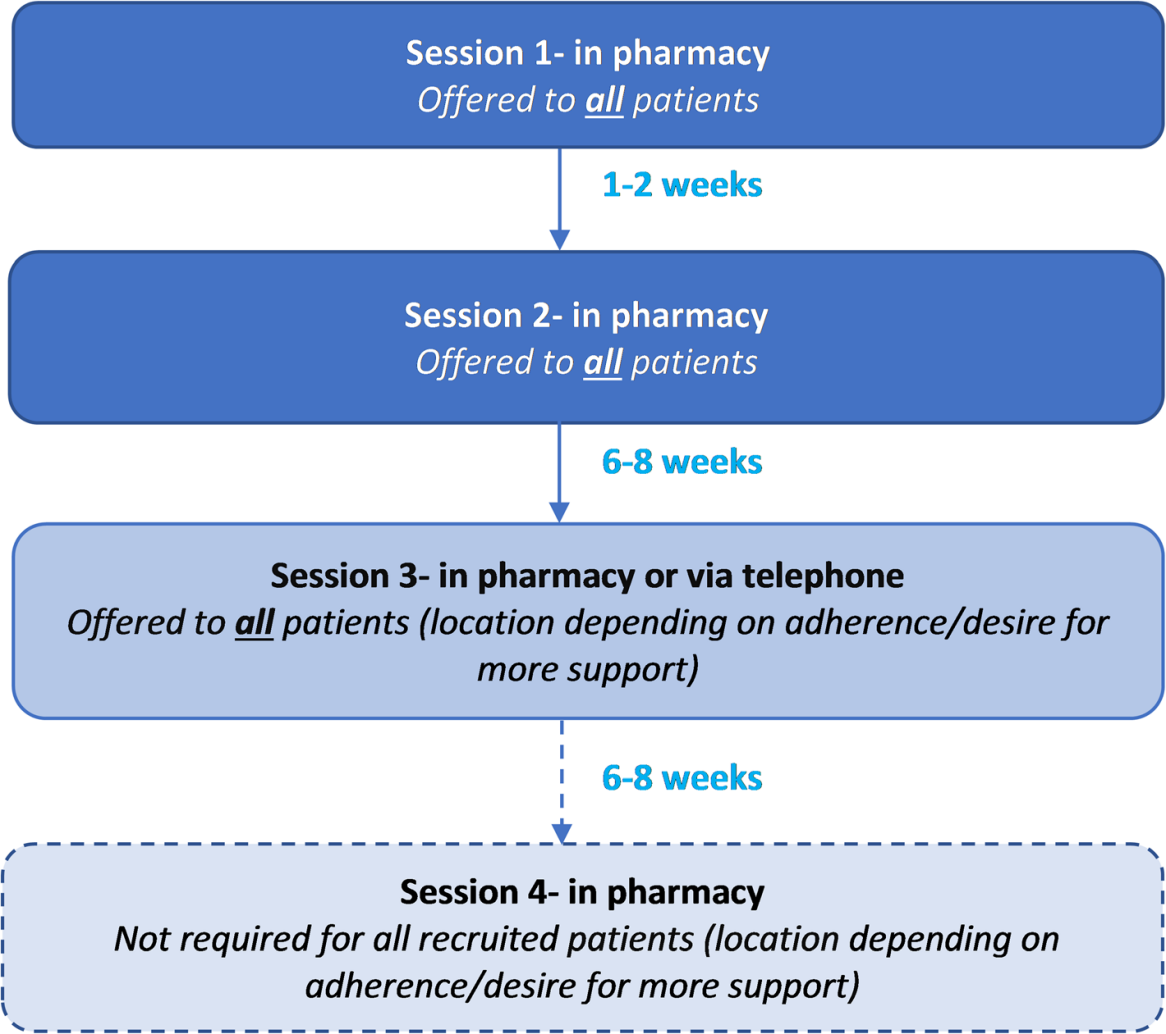
Overview of sessions and timings in the S-MAP intervention

The intervention was designed to be delivered over three to four sessions depending on the patient’s level of adherence and desire for additional support (see Fig. 1). The app was designed to guide the pharmacist in tailoring the number and type of sessions. Adherence was re-assessed at each follow-up session using MARS-5 [22] and one item adapted from Lu et al. [23].

**Fig. 2:**
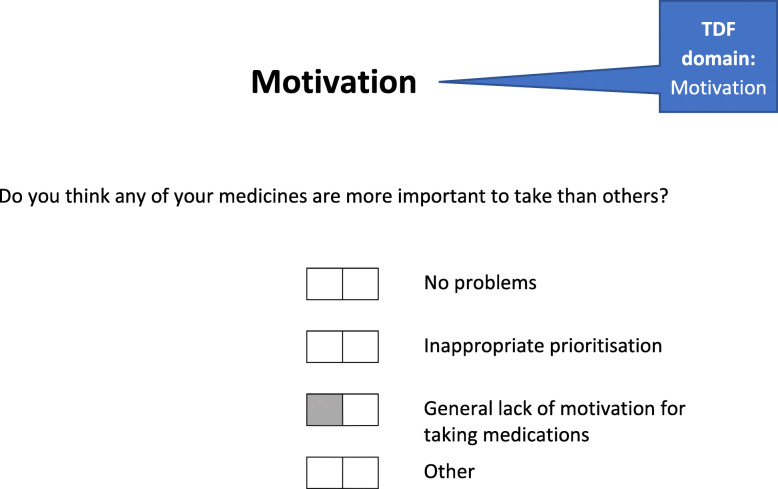
An example of an adherence assessment question in the S-MAP web application

**Fig. 3:**
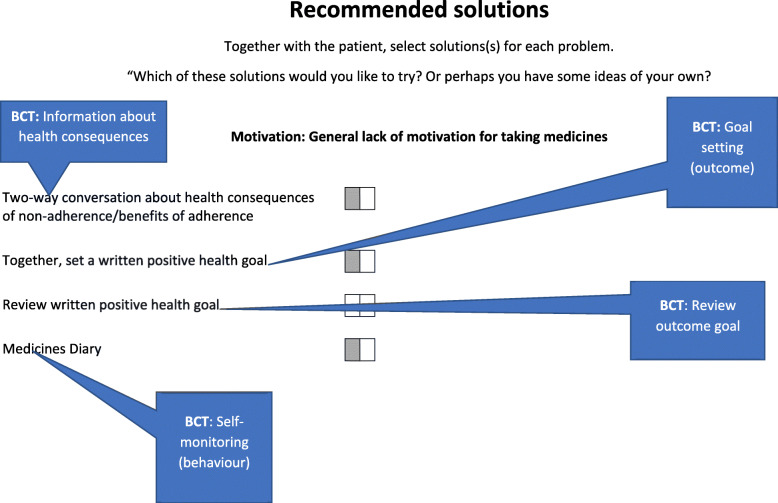
An example of recommended solutions displayed for an identified adherence problem in the S-MAP web application

At session 1, an adherence assessment (BCT: problem solving) was undertaken, guided by the app, to explore a range of barriers to adherence that were identified as important in our previous research [11] such as knowledge, routines, forgetfulness and motivation (see example in Fig. 2). Each identified adherence problem was then automatically linked to a range of potential solutions that reflect specific BCTs (see example in Fig. 3). Together, patients and pharmacists selected the most appropriate solutions to address each of the identified problems. All patients were also offered a medication diary at session 1 to encourage them to self-monitor their medication-taking behaviour and identify any instances of non-adherence over the course of the intervention period which they could then discuss with the pharmacist at follow-up sessions.

At follow-up sessions, a review of the medication diary was undertaken by the pharmacist and feedback given if the diary was completed. Alternative (or additional) solutions could be recommended if adherence had not improved and/or if the patient requested more support. The data recorded on the app were saved at the end of each session and pharmacists could review this in advance of the next session. Space for recording brief notes during or after the sessions was also available. Further details of the intervention sessions and content can be found in the study protocol [17].

### Training package

The interactive 1-day training workshop included an introduction to the study, study procedures (e.g. recruitment), information on the intervention, video demonstrations of the app, a practice session using the app and role-play activities focusing on new skills required to deliver BCTs such as goal-setting and action planning. The training workshops were audio-recorded with pharmacists’ permission and pharmacists completed a feedback survey. These data will be analysed and reported as part of a separate process evaluation.

### Outcome data collection

Outcome data were collected at baseline/pre-session 1 (questionnaires administered by pharmacy staff), and again at 6 months’ post-baseline (questionnaires administered by research team).

### Primary outcome

The primary outcome was medication adherence across multiple medications. This was measured using two methods which included both subjective and objective measures. This multi-method approach is currently recommended due to the lack of a single gold standard measure for medication adherence [30].

#### Medication possession ratio and daily polypharmacy possession ratio

Using dispensing data obtained from participating community pharmacy PMR computer systems, the Medication Possession Ratio (MPR) and Daily Polypharmacy Possession Ratio (DPPR) were calculated for each patient [31, 32]. MPR is the most widely used measure of adherence based on pharmacy dispensing data [32]. MPR can be calculated by dividing the number of days of medication supply obtained in the observation window (minus the number of days supplied in the last dispensing episode) by the number of days between the first and last dispensing episode in the observation window [31]. At least two dispensing instances in the observation window are therefore required for the calculation of MPR. In the current study, the MPR was capped at 1 (i.e. 100%) for each medication and an average MPR (multiplied by 100 to give a %) for all regular medications calculated for each patient. However, MPR may either over- or under-estimate adherence to polypharmacy regimens, for example, because it does not consider overlapping supplies of medications [33]. The DPPR is a relatively new measure of adherence based on dispensing data, that aims to overcome some of the limitations with MPR as it ‘…accounts for the specificity of polypharmacy’ including overlapping supplies [33]. The DPPR reflects the proportion of time that multiple medications are available in the observation window. This is calculated by looking at each day in the observation window and considering whether the patient has each regular medication available or not. The proportion of medicines available for each day in the observation window is then summed and divided by the number of days in the observation window to produce the DPPR (this is then multiplied by 100 to give a %).

The observation windows for assessment of both MPR and DPPR in this study were the 6 months pre-session 1 (window 1) and 6 months post-session 1 (window 2). For the DPPR, accumulated unused oversupply of medicines dispensed in the 6-month period before each observation window could be carried over into the observation window. Data were collected for the 12 months pre-session 1 to facilitate this carryover into window 1. DPPR and MPR were calculated using the statistical programme R and AdhereR package [34]. MPR was calculated using the CMA3 (Continuous Medication Availability) function in AdhereR [34] and DPPR was calculated using code supplied by Dr. Samuel Alleman (personal communication), who co-developed the DPPR code for AdhereR (CMA-polypharmacy function). To ensure the code was functioning correctly, manual calculations of both DPPR and MPR were performed and comparisons made with the R computations.

Only medications prescribed on a regular basis (> 3 months’ supply) were included. Medications that are commonly recommended for short periods of time and/or for symptomatic relief were excluded (e.g. pain-relieving agents, laxatives, hypnotics, anxiolytics, antihistamines). Medications that were prescribed with ‘when required’ or variable dosing directions (e.g. take 1 or 2 tablets daily) or formulations where dosing is difficult to estimate (e.g. creams, insulin) were also excluded. Medications with simple dosage changes (e.g. increased from 2 to 4 mg strength) were included; however, more complex switches such as changes between combination products and individual medications were excluded [33, 35].

#### Self-reported adherence

Self-reported adherence was measured using MARS-5 [22] and one item adapted from Lu et al. [23]. Permission for use of MARS-5 was granted by the developer (Professor Robert Horne). At baseline, these measures were initially administered by pharmacy staff as part of the screening process; however, following the amendments to screening procedures, these measures were administered following recruitment (i.e. before session 1) along with secondary outcome measures (see below).

### Secondary outcomes

Secondary outcome measures included health-related quality of life (HRQOL), measured using the EQ-5D-5L questionnaire (UK version), and unplanned hospital admissions, resulting in an overnight stay which was measured via a self-report tool developed specifically for the purposes of this study. The EQ-5D-5L consists of two sections: a five-item questionnaire and a visual analogue scale (EQ-VAS) [36]. EQ-5D-5L utility index scores were calculated using question responses and a mapping function developed by van Hout et al. [37] as recommended currently by the UK National Institute for Health and Care Excellence (NICE) [38]. Index scores less than 0 are described as being worse than death and a score of 1 indicates a state of perfect health. These measures were administered at baseline (pre-session 1) by the pharmacist/trained pharmacy support staff and again at 6 months’ post-baseline (via postal questionnaire administered by the research team). Patients were sent reminder copies of the questionnaire and non-responders contacted via telephone (where contact numbers were provided). Information on unplanned hospital admissions was also cross-checked with GP-held records which community pharmacists obtained via telephone from the patient’s GP practice.

### Data analysis

For all descriptive statistics (e.g. patient age, MPR, MARS-5), the mean and standard deviation (SD) are presented where data are approximately normally distributed, and the median and interquartile range (IQR) reported where data are non-normally distributed. For the dispensing data (MPR, DPPR), self-reported adherence (MARS-5, Lu item) and HRQOL (EQ-5D-5L utility index and EQ-VAS) scores pre- and post-session 1, confidence intervals (95%) and effect sizes (*r*) are also provided. Effect sizes (*r*) have been presented to give an indication of the magnitude of change between pre- and post-session 1 scores [39]. An effect size (*r*) of ± 0.1 is deemed to be a small effect size, ± 0.3 a medium effect size and ± 0.5 a large effect size [40]. Due to the small numbers of unplanned hospital admissions observed during the study period, the total numbers of admissions (and number of patients with admissions) have been presented. Patients were excluded from an outcome analysis if they had missing data at pre- or post-session 1. Analysis was conducted using SPSS (IBM Corp. Released 2019. IBM SPSS Statistics for Windows, Version 26.0. Armonk, NY: IBM Corp.)

### Progression criteria

Explicit a priori progression criteria, published in the study protocol paper [17], have been used to determine whether to proceed to a larger c-RCT to assess the effectiveness of the enhanced S-MAP intervention [41]. Cut-off points were developed based on work published by Borelli et al. [42], which indicates that high fidelity is when ≥ 80% of the criteria are met (‘Stop’), medium fidelity is when 50% of the criteria are met (‘Amend’) and low fidelity is when < 50% of the criteria are met (‘Stop’). Data to support the decisions for progression criteria concepts related to training and intervention fidelity, including in-depth qualitative analyses, will be reported in a separate process evaluation paper.

### Ethical approval, reporting and patient/public involvement

The study was granted ethical approval by the Office of Research Ethics Committees for Northern Ireland (REC reference: 17/NI/0193) and the study protocol published in advance [17]. This study has been reported in line with the Consolidated Standards of Reporting Trials (CONSORT) extension for reports of randomised pilot and feasibility studies as recently recommended by Lancaster and Thabane [43]. A completed CONSORT checklist can be found in Additional file 3. Two patients and two community pharmacists from NI and London formed part of a Patient and Public Involvement (PPI) advisory group that provided advice to the research team during the study.

## Results

### Pharmacy sampling, recruitment and retention

Twelve community pharmacies were recruited over a 3-month period (April-June 2018), six from the five HSCT areas in NI and one from each of the six selected CCGs in London. From the 120 letters posted, 12 reply slips expressing interest were returned [7 in NI, 5 in London; overall response rate (letter) = 10%]. From those who returned reply slips, eight pharmacies were recruited (3 in NI, 5 in London; overall recruitment rate (letter) = 7%). Reasons for non-participation/ineligibility from the posted invitations included the lack of a suitable consultation area (*n* = 1) and difficulty with arranging an initial site visit (*n* = 1). No specific reason was provided by one pharmacy and one reply slip was received after recruitment targets were met. Pharmacies who did not return reply slips were contacted via telephone until the recruitment target of 12 was achieved. Seven non-responding pharmacies in NI and 14 pharmacies in London (total *n* = 21) from CCGs/HSCT areas where no pharmacies had been recruited (including a range of different types of pharmacies) were contacted via telephone. Six pharmacies in NI and 7 in London (total *n* = 13) expressed potential interest at this stage [overall response rate (follow-up phone call) = 62%] and were sent additional study information. Four further pharmacies were recruited using this approach [3 in NI, 1 in London; overall recruitment rate (follow-up phone call) = 19%], thereby achieving the recruitment target. Reasons for non-participation from the telephone follow-ups included: insufficient time (*n* = 8), difficulties with arranging locum cover (*n* = 1) and the need for head office to approve participation (*n* = 1). No specific reason for non-participation was provided by one pharmacy and six pharmacies were no longer required as recruitment targets were met. At the end of the 12-month study period, 11 of the 12 recruited pharmacies had been retained. One pharmacy in London withdrew from the study prior to the recruitment of patients due to insufficient time.

### Pharmacy and staff characteristics

In NI, four pharmacies were independently owned (1-3 stores) and two were part of large chains (10+ stores). NI pharmacies were located in areas with NI MDM scores of 55, 176, 238, 342, 390, 431 (where 1 = most deprived area, 890 = least deprived area in NI) with two pharmacies located in urban areas and four pharmacies located in rural areas [19]. In London, five pharmacies were independently owned (1-5 stores) and one was part of a small chain (6-99 stores). London-based pharmacies were located in areas with IMD decile scores of 2, 2, 5, 6, 9 and 9 (where 1 = most deprived area, 10 = least deprived area in London) [18] and were located in both urban and suburban areas. Seven pharmacies had one full-time equivalent (FTE) pharmacist staff member working on an average weekday and three pharmacies had two FTE pharmacists. One pharmacy had an additional pharmacist working 1 day per week. Pharmacies had a median of three (IQR: 2.3-4.0) FTE pharmacy support staff such as dispensing staff and medicines counter assistants. On a typical weekday, the number of prescriptions dispensed by pharmacies was 100-199 (*n* = 2), 200-299 (*n* = 4), 300-399 (*n* = 3), 400-499 (*n* = 1), 600+ (*n* = 1). Data on staffing levels and prescription items were not collected from the pharmacy that dropped out during the study.

Fifteen community pharmacists, from the 12 recruited pharmacy sites, took part in the study. Three sites had two pharmacists participating at each site, and six sites each had one support staff member (including four pre-registration pharmacists) who assisted with study procedures. Recruited pharmacists (*n* = 14) working in pharmacies that were retained in the study had been practising for a median of 15.5 years (IQR: 10.8-29.3). Three pharmacists were pharmacy owners/proprietors, seven were pharmacy managers and four were support/second pharmacists. Nine pharmacists (64.3%) had undertaken previous training in supporting patients with medication adherence and two pharmacists were trained as independent prescribers.

### Pharmacy staff training

Recruited pharmacists attended training workshops in August and September 2018. Seven pharmacists and one support staff member (pre-registration pharmacist) from the six pharmacy sites in NI attended the Belfast workshop. In London, six pharmacists from five of the pharmacy sites attended the workshop. The training was delivered over approximately 6 h by the same two members of the research team (DP, EC). The online distance learning Moodle® package was completed by five support staff members and one pharmacist in London and one support pharmacist in NI who were unable to attend the workshops. A member of the research team then visited each pharmacy site to discuss any queries.

### Patient screening, recruitment and retention

Patient screening and recruitment took place between August 2018 and July 2019. Screening and recruitment paperwork was completed for 104 patients in NI and for 36 patients in London. Information on screened patients, including rates of non-adherence and recruitment rates, can be found in Table 1. Feedback from pharmacists in both NI and London during the study indicated that they did not complete the screening paperwork for all of the patients that they screened due to time constraints.

**Table 1:** Data from screening and recruitment paperwork completed by pharmacists

	NI	London	Total
**Initial screening procedures**^a^			
Number of screening forms completed	74	24	98
Number of patients who completed the screening questionnaire (%)	52 (70.3)	22 (91.7)	74 (75.5)
Non-adherence rate^b^, % (number of eligible non-adherent patients)	46.2 (24); missing = 2	81.8 (18); missing = 0	56.8 (42); missing = 2
Recruitment rate^c^, % (number recruited)	62.5 (15)	77.8 (14)	69.0 (29)
**Modified screening procedures**^d^			
Number of screening forms completed	30	12	42
Non-adherence rate^e^, % (number of eligible non-adherent patients)	73.3 (22)	83.3 (10)	76.2 (32)
Recruitment rate^c^, % (number recruited)	95.5 (21)	100 (10)	96.9 (31)
Total number of screened patients (%)	104 (74.3)	36 (25.7)	140
**Characteristics of recruited patients**			
Total number of patients recruited (%)	36 (60.0%)	24 (40.0%)	60
Mean age (± SD; range)	70.1 (± 8.5; 50-85); missing = 0	68.3 (± 8.8; 50-84); missing = 2	69.5 (± 8.6; 50-85); missing = 2
Female, *n* (%)	23 (63.9)	14 (58.3)	37 (61.7)
Median number of prescribed medications (IQR; range)	7 (6-8; 4-13); missing = 1	8 (5-9; 3-15); missing = 1	7 (5.8-8.3; 3-15); missing = 2
Median number of prescribed regular medications (IQR; range)	6 (5-7; 3-12); missing = 1	6 (5-7; 3-9); missing = 1	6 (5-7; 3-12); missing = 2
Median number of prescribed non-regular^f^ medications (IQR; range)	0 (0-1; 0-4); missing = 1	1 (0-3; 0-7); missing = 1	1 (0-2; 0-7); missing = 2

^a^Included a screening questionnaire (Lu item and MARS-5) to assess adherence. Patients aged 65+ were eligible to take part

^b^Number of eligible non-adherent patients from the total number who completed adherence screening questionnaire

^c^Number of patients recruited from the total number of eligible non-adherent patients

^d^Adherence assessment completed using the PMR or via general discussions with the patients. Age limit lowered to 50+ years

^e^Number of eligible non-adherent patients from the total number of patients screened

^f^Includes medications used on a short-term basis or generally for symptomatic (when required) use

In total, using both screening approaches, 60 patients were recruited into the study (36 in NI and 24 in London). Pharmacies recruited between 0 and 10 patients each (median: 4; IQR: 1.3-9.8) with three pharmacies recruiting 10 patients and two pharmacies recruiting 8 and 9 patients each. The mean age of recruited patients was 69.4 (SD: ± 8.5; range: 50-85) years and 37 participants (61.7%) were female. Additional information is provided in Table 1.

**Fig. 4:**
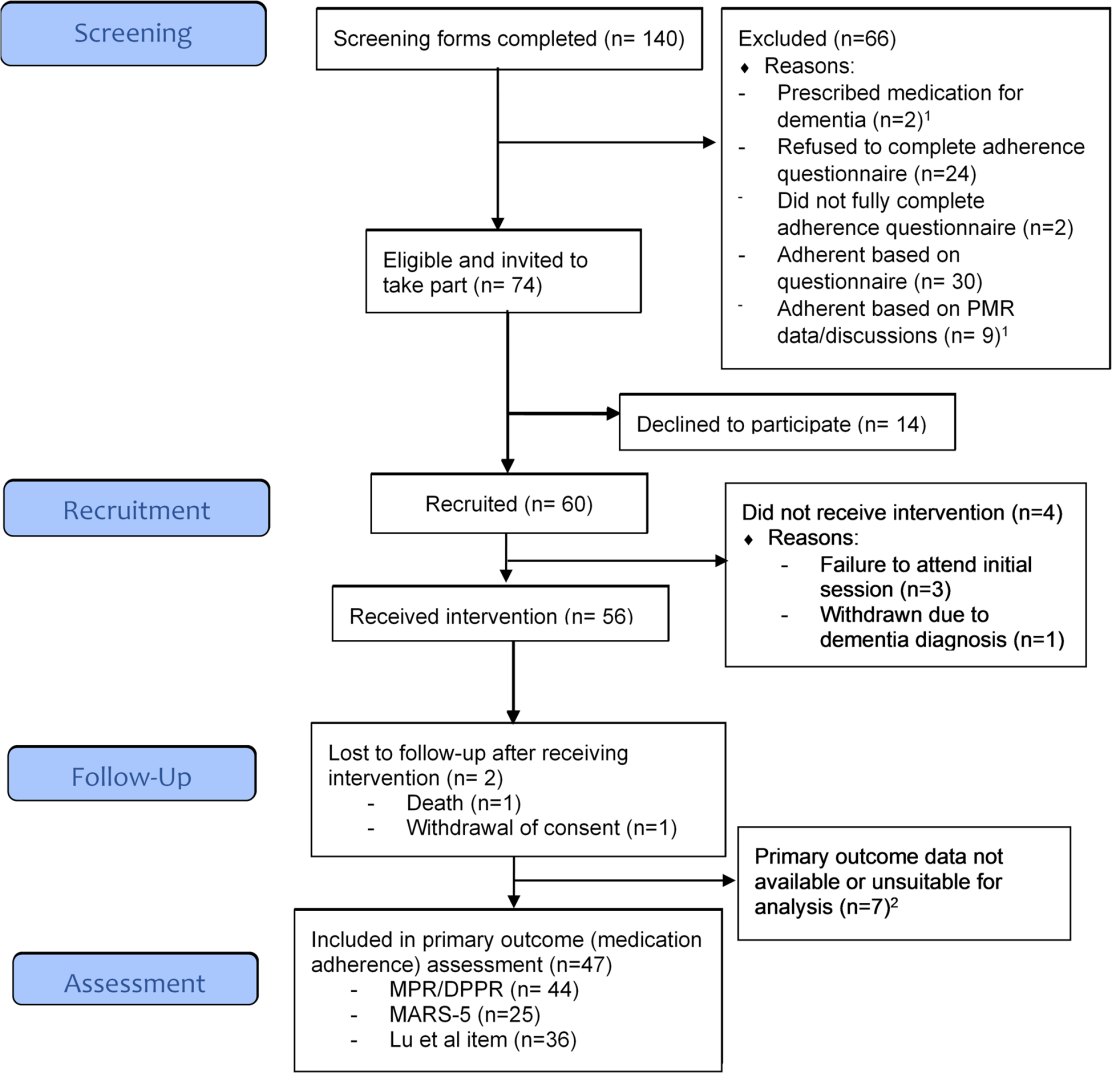
Participant flow diagram for the S-MAP study. ^1^One patient was ineligible for two reasons: prescribed medications for dementia and adherent based on PMR data/discussions; ^2^Reasons included non-completion of the baseline questionnaire, non-completion of 6-month follow-up questionnaire, unable to collect dispensing data due to COVID-19 pandemic and/or dispensing data not suitable for analysis due to instalment (weekly) dispensing

During the study, there were six patient withdrawals (3 in NI, 3 in London). Reasons included: death (*n* = 1), dementia diagnosis (*n* = 1), patient withdrawal of consent (*n* = 1) and failure to attend the initial S-MAP session (*n* = 3). In addition, seven patients did not have any primary outcome data suitable for analysis (i.e. dispensing data or self-report adherence data) (see ‘Primary outcomes’ section). Therefore, 47 of the 60 recruited patients were deemed to be retained in the study, giving an overall retention rate of 78.3%. A participant flow diagram is presented in Fig. 4.

### Intervention delivery

Ten of the 15 participating pharmacists, from nine pharmacy sites, conducted sessions with 56 patients. In total, 155 sessions were delivered between September 2018 and October 2019 (99 in NI and 56 in London). Of these, 118 (76.1%) sessions were conducted in the pharmacy and 37 sessions were completed via telephone (23.9%). The number of sessions attended by patients included none (*n* = 4), one (*n* = 6), two (*n* = 4), three (*n* = 43) and four (*n* = 3). Four patients brought along a family member to the sessions and the GP was contacted on only one recorded occasion to confirm the accuracy of the patient’s prescribed medication list. The duration of sessions was automatically recorded by the app upon clicking the ‘complete’ button (Table 2). However, some pharmacists reported that data were occasionally entered retrospectively (e.g. following completion of the session without the app or forgetting to save the session). The recorded durations of some sessions (Table 2) may therefore underestimate the true session length.

**Table 2:** The duration of sessions in the S-MAP intervention (recorded by the app)

Session number	Location	***N*** (missing^a^)	Median session duration in mm:ss^b^(IQR)	Range
1	Pharmacy	53 (3)	15:18 (08:41-25:37)	03:24-52:21
2	Pharmacy	49 (1)	07:44 (02:40-16:48)	00:45-38:25
3	Telephone	36 (1)	01:37 (00:46-04:01)	00:22-21:08
	Pharmacy	9 (0)	11:44 (08:01-15:53)	02:27-30:48
4	Pharmacy	3 (0)	03:23 (NA^c^)	01:52-20:39

^a^Error with time recording—data excluded

^b^Minutes: seconds

^c^Unable to calculate Interquartile range (IQR) due to low numbers

The median number of days between sessions 1 and 2 was 30.5 days (*n* = 50; IQR, 15.0-69.3; recommended, 7-14), between sessions 2 and 3 was 60 days (*n* = 46; IQR, 48.5-88.5; recommended, 42-56) and between sessions 3 and 4 was 49 days (*n* = 3; IQR, unable to calculate; recommended, 42-56).

During session 1, an adherence assessment was conducted for each patient (*n* = 56). Barriers to adherence were identified during this session for all but one patient (who chose to withdraw from the study following the first session). A median of two adherence barriers was identified per patient (IQR, 2-4). A wide range of barriers to adherence were identified including knowledge issues (*n* = 23), daily routine/organisation issues (*n* = 17), practical barriers (*n* = 12), lack of support from others (*n* = 5), belief-based barriers (*n* = 32) and motivational barriers (*n* = 8). Further information on the types of barriers identified during the assessment are presented in Table 3. Pharmacists were also given the opportunity to record any additional barriers that were not listed as options on the app. All additional barriers recorded in this section could have been categorised into barriers already listed on the app (*n* = 3).

**Table 3:** Barriers to adherence identified during the adherence assessment conducted during session 1

Barriers identified	Number of patients
**Knowledge** (*TDF domain: Knowledge*)	
Knowledge issues	23
**Routine/organisation** (*TDF domains: Nature of the behaviours; behavioural regulation; memory, attention and decision processes*)	
Difficulties ordering supplies of medications	11
No clear routine for taking medications	9
**Total number of patients with routine/organisation barriers**^a^	**17**
**Forgetting** (*TDF domains: Memory, attention and decision-making processes; environmental context and resources; behavioural regulation*)	
Occasional forgetfulness	30
Frequent forgetfulness	6
Forgets when away from home	6
**Total number of patients with forgetting barriers**^a^	**39**
**Practical difficulties** (*TDF domain: Environmental context and resources*)	
Dexterity/administration issue	3
Packaging/formulation issue	6
Unable to read/understand labels	2
Difficulty swallowing	5
Regimen too complex	1
**Total number of patients with practical barriers**^a^	**12**
**Social support from others** (*TDF domain: Social influences*)	
More support required	5
**Beliefs/intentional non-adherence** (*TDF domains: Beliefs about consequences*)	
Reasons for missed doses	
General concerns about medications	10
Concerns about generic medicines	1
Experiencing side effects	11
Lack of symptoms	5
Unaware of health consequences	7
Worried about side effects	9
Uncertain of benefits	11
**Total number of patients who reported missed doses**^a^	**32**
**Motivation** (*TDF domain: Motivation and goals*)	
Inappropriate prioritisation	4
General lack of motivation for taking medicines	4
**Total number of patients with motivation barriers**^a^	**8**

^a^Patients may have had multiple different types of barriers identified in this category

Following identification of barriers, pharmacists delivered a wide range of tailored adherence solutions as recommended by the app. One pharmacist who conducted a session with only one patient did not identify any adherence barriers and so did not deliver any solutions. Therefore, nine pharmacists delivered solutions to 55 patients. In total, 265 solutions were delivered during the sessions with a median of five solutions per patient (IQR: 2-7). Of these, 129 were practical/social support-based solutions, with a median of two per patient (IQR: 1-3) and 57 were belief/motivation/goal-based solutions, with a median of one per patient (IQR: 0-2). The majority of solutions (*n* = 244; 92%) were delivered at session 1. Further details of the types of solutions delivered during the sessions are detailed in Table 4.

**Table 4:** Adherence solutions delivered during sessions as part of the S-MAP intervention (as recorded by pharmacists on the app)

Adherence solution^a^	Behaviour change techniques (BCT) [29]	Number of pharmacists who delivered the solution^b^	Number of patients who received the solution^c^
**Self-monitoring solutions**			
Medication diary accepted	Self-monitoring of behaviour	9	31
Travel-size version of diary supplied		1	1
Diary completed and reviewed at session 2	Self-monitoring of behaviour; feedback on behaviour	7	25
**Knowledge solutions**			
Education/knowledge issues addressed	Information about health consequences^d^	7	23
**Practical/social support solutions**			
Store medicines in visually prominent place	Restructuring the physical environment	7	25
Large print labels		1	1
Link medications to common routine	Prompts/cues	8	25
Reminder in own travel itinerary		2	3
Mobile phone reminders		4	6
Mark-reorder dates on diary	Prompts/cues; self-monitoring	1	4
Synchronise medication supply with GP	Social support (practical)	4	7
Practical support—family/friends		3	4
Pharmacy help with prescription re-ordering		4	9
Pharmacy to collect prescriptions		3	7
Pharmacy delivery service		3	3
Pharmacy supplied MDS	Adding objects to the environment	2	3
Purchase pill reminder box		6	7
Alternative packaging		4	4
Physical aids		0	0
Swallowing tips	Instruction on how to perform the behaviour	4	5
Simple advice to improve administration		3	3
Discuss options with prescriber	NA	5	13
**Number of patients who received practical/social support solutions**			***46/55***
**Number of pharmacists who delivered practical/social support solutions**		***9/9***	
**Belief/motivation/goal solutions**			
Voicing concerns about medicines leaflet	Information about health consequences^d^	5	11
Generic medicines leaflet		0	0
Two-way conversation about benefits of adherence/consequences of non-adherence		6	18
Strategies to manage mild side effects	NA	4	8
Set positive health goal	Goal setting (outcome)	1	2
Set adherence goal	Goal setting (behaviour)	3	8
Develop action plan	Action planning	3	8
Review positive health goal	Review outcome goal	0	0
Review adherence goal	Review behaviour goal	2	2
**Number of patients who received belief/motivation/goal solutions**			**30/55**
**Number of pharmacists who delivered belief/motivation/goal solutions**		**8/9**	

^a^Some solutions were repeated/delivered again at follow-up sessions but these have only been counted once

^b^Nine pharmacists delivered solutions to patients during sessions

^c^Fifty-five patients had barriers identified and solutions delivered

^d^Included information on the importance of raising concerns, managing side effects, the importance of taking medicines as prescribed and only stopping medicines after consulting the prescriber

### Primary and secondary outcomes

Six-month follow-up data for the primary and secondary outcomes were collected between March 2019 and March 2020. The proportion of missing data across both primary and secondary outcome measures was 30.1% (Additional file 4).

#### Primary outcomes: adherence (MPR, DPPR and self-report)

Full dispensing records covering the six months pre- and post-session 1 were collected for 52 patients. Full dispensing records were not available for the six patient withdrawals noted previously and records could not be collected for two patients due to contextual issues in the community pharmacy setting (i.e. the COVID-19 pandemic that arose during the final period of data collection). From the 52 records obtained, 44 patients (73.3%) had dispensing data that were suitable for analysis. Dispensing data were not suitable for analysis for six patients who had medications dispensed on a weekly basis. As pharmacies have different processes for prescription ordering and the production of dispensing labels/records for these types of patients, these were not deemed to be an accurate representation of the patient’s medication possession and were therefore excluded. Two patients were excluded as they had fewer than two medications suitable for analysis.

Baseline questionnaire data, including self-reported adherence data (Lu item, MARS-5), were collected for 51 of the 60 recruited patients. Pharmacy staff did not administer baseline questionnaires to nine recruited patients. Six-month follow-up questionnaires were mailed by the research team to 54 of the 60 recruited patients (six patients withdrew from the study prior to this follow-up period). In total, 43 out of the 54 follow-up questionnaires were returned (i.e. 79.6% response rate). Thirty-seven patients (61.7%) had both baseline and 6-month follow-up self-reported adherence data suitable for analysis.

Median scores and effect sizes (*r*) for DPPR, MPR and self-reported adherence measures (Lu item, MARS-5) in the 6-months pre- and 6-months post-session 1 are presented in Table 5. The median number of medications eligible for the DPPR and MPR calculations per patient was five (IQR: 4-6).

**Table 5:** DPPR, MPR, Lu item, MARS-5, EQ-5D-5L utility and EQ-VAS scores pre- and post-session 1

	Number of patients	Pre-session 1 median (IQR)	95% confidence intervals for pre-session 1 median	Post-session 1, median (IQR)	95% confidence intervals for post-session 1 median	***Z***^a^	Effect size (***r***)^b^
**Primary outcome: dispensing data measures (DPPR, MPR)**							
DPPR (%)	44	85.2 (72.0-96.7)	73.9-92.9	93.8 (84.8-97.8)	86.8-96.7	−3.80	−0.41
MPR (%)	44	93.8 (82.8-100.0)	88.9-97.5	94.6 (87.8-97.8)	89.7-97.5	−1.28	−0.14
**Primary outcome: self-reported adherence**							
Lu item (%)	36	80 (60-80)	60-80	100 (80-100)	80-100	−3.45	−0.41
MARS-5 total	25	22 (20-23.5)	21-23	24 (24-25)	24-25	−3.12	−0.44
**Secondary outcome: EQ-5D-5L**							
EQ-5D-5L- Utility index	30	0.73 (0.52-0.84)	0.53-0.80	0.74 (0.58-0.84)	0.65-0.77	−0.91	−0.12
EQ-5D-5L-VAS	35	75 (50-83)	60-80	75 (60-85)	65-80	−0.12	−0.01

Key: *DPPR* daily polypharmacy possession ratio; *MARS-5* Medication Adherence Report Scale; *MPR* medication possession ratio; *IQR* interquartile range

^a^Test statistic for Wilcoxon signed-rank test

^b^Effect size (*r*) calculated using *r* = Z/√N; where *Z* is the z score for the Wilcoxon signed-rank test and *N* is the total number of observations. −0.1 indicates a small effect size, −0.3 indicates a medium effect size and −0.5 indicates a large effect size

#### Secondary outcome: quality of life (EQ-5D-5L)

Thirty-five patients completed the EQ-5D-5L in both the pre- and post-session 1 questionnaire. Median scores and effect sizes (*r*) for the EQ-5D-5L utility index and EQ-VAS in the 6-months pre- and 6-months post-session 1 are presented in Table 5.

#### Secondary outcome: Unplanned hospitalisations

Self-reported and GP-reported pre- and post-session 1 data on unplanned hospitalisations were available for 23 and 47 patients, respectively. Full 6-month pre- and post-session 1 data were not available for the six patients who withdrew, so these were excluded from this analysis. GP-reported hospitalisations data could not be collected for one pharmacy site (*n* = 7 patients) due to the COVID-19 pandemic.

In the 6 months’ pre-session 1, there were four self-reported unplanned hospital admissions (from three patients) and six GP-reported admissions (from four patients). In the 6 months’ post-session 1, there were two self-reported admissions (from two patients) and one GP-reported admission.

### Progression criteria

Following assessment of the a priori progression criteria, nine concepts met the ‘Go’ criterion (see Table 6) and three concepts (‘Patient recruitment’, ‘Patient retention’ and ‘Missing data’) met the ‘Amend’ criteria. None of the decision criteria indicated that a further evaluation of S-MAP should not proceed. The concept, ‘Acceptability of intervention to pharmacists’, was not assessed for the purposes of progression decision-making. The detailed qualitative feedback interviews with community pharmacists that were intended to be used for this progression concept did not ask participants for an overall binary evaluation of the intervention (is the intervention acceptable?—yes or no). Alternatively, these interviews focused on exploring the degree of acceptability of specific components of the S-MAP intervention (e.g. the app, the medication diary, barrier identification, selecting solutions). The qualitative data on intervention acceptability will be reported in-depth as part of the linked process evaluation and published separately. ‘Fidelity of pharmacist training package receipt’ was assessed solely using a post-workshop feedback survey. Data obtained from audio-recordings of pharmacist workshops did not contribute to the decision-making process for this concept, as was originally planned, due to the low quality of audio-recordings which did not consistently pick up each individual voice, therefore the data required for the analysis were not available.

**Table 6:** Final decisions for each of the progression criteria (‘Stop’, ‘Amend’, ‘Go’) for the S-MAP pilot study

Concept	Data source(s)	Progression criteria			Final decision
		Stop (unless there are clear and modifiable contextual or design issues that account for this^a^)	Amend	Go	
Pharmacy recruitment	Recruitment records held by research team	If ≤ 5 pharmacies are recruited within 8 months	If 6-9 pharmacies are recruited and/or it takes longer than predicted (> 4-6 months)	If ≥ 10 pharmacies are recruited to take part in ≤ 4 months	**Go**: 12 pharmacies were recruited within 3 months.
Pharmacy retention	Retention records held by research team	If ≤ 49% of pharmacies are retained for the required period	If 50%-79% of pharmacies are retained for the required period	If ≥ 80% of pharmacies are retained for the required period	**Go:** 92% of pharmacies (i.e. 11) were retained until the end of the study period.
Patient recruitment	Study documentation completed by pharmacy staff	If ≤ 59 patients are recruited within 6 months^b^ or alternatively^c^ if ≤ 49% of pharmacies achieve a monthly recruitment rate of 2 patients per month for any 3 consecutive months	If 60-95 patients are recruited within 6 months^b^ or alternatively^c^ if 50-79% of pharmacies achieve a monthly recruitment rate of 2 patients per month for any 3 consecutive months	If ≥ 96 patients are recruited within 6 months^b^ or alternatively^c^ if ≥ 80% of pharmacies achieve a monthly recruitment rate of 2 patients per month for any 3 consecutive months	**Amend:** 60 patients were recruited within 12 months^b^ (extension to 12 months was made following amendments to screening/recruitment procedures).
Patient retention	Study documentation completed by pharmacy staff	If ≤ 49% of patients are retained for the required period	If 50%-79% of patients are retained for the required period	If ≥ 80% of patients are retained for the required period	**Amend:** 78.3% of patients were retained in the study (i.e. had either dispensing or self-reported adherence data available for primary outcome analysis). 73.3% of patients had data available for dispensing data analysis and 61.7% of patients had self-reported adherence data available for analysis.
Missing Data	Data collected during the study (questionnaires, dispensing data)	If ≥ 50% of the main outcome data are missing	If 21-49% of the main outcome data are missing	If ≤ 20% of the main outcome data are missing	**Amend:** 30.1% of primary and secondary outcome data were missing (see additional file 4).
Fidelity of pharmacist training package: delivery	Audio-recordings of pharmacist workshops^d^	If ≤ 49% of planned training components are delivered by the researchers	If 50-79% of planned training components are delivered by the researchers	If ≥80% of planned training components are delivered by the researchers	**Go:** 87% (13/15) of the planned training components (i.e. BCTs) included in the training practice were delivered by the researchers^d^
Fidelity of pharmacist training package: receipt	Post-workshop feedback survey^d^	If ≤ 49% of pharmacists report that they feel prepared to take part in the study	If 50-79% of pharmacists report that they feel prepared to take part in the study	If ≥ 80% of pharmacists report that they feel prepared to take part in the study	**Go:** 93% (*n* = 13) of pharmacists reported feeling ‘very prepared’ (*n* = 8) or ‘somewhat prepared’ (*n* = 5) in the post-workshop feedback survey^d^
	Audio-recordings of pharmacist workshops^d^	If ≤ 49% of delivered training components are received by pharmacists as intended	If 50-79% of delivered training components are received by pharmacists as intended	If ≥ 80% of delivered training components are received by pharmacists as intended	This data source did not contribute to the decision-making process for this concept (see reasons for this in the results section).
Acceptability of pharmacist training day	Post-workshop feedback survey^d^	If ≤ 49% of pharmacists report that the training day was acceptable	If 50-79% of pharmacists report that the training day was acceptable	If ≥ 80% pharmacists report that the training day was acceptable	**Go:** 100% of pharmacists (*n* = 14) rated the training day as completely acceptable (*n* = 12) or acceptable (*n* = 2) in the post-workshop feedback survey^d^
Acceptability of intervention to pharmacists	Post-intervention delivery qualitative interviews^d^	If ≤ 49% of pharmacists report that the intervention was acceptable	If 50-79% of pharmacists report that the intervention was acceptable	If ≥ 80% pharmacists report that the intervention was acceptable	This concept was not assessed (see reasons for this in the ‘Results’ section).
Acceptability of intervention to patients	Post-intervention delivery feedback survey^d^	If ≤ 49% of patients report that the intervention is acceptable	If 50-79% of patients report that the intervention is acceptable	If ≥ 80% of patients report that the intervention is acceptable	**Go:** 88.6% (*n* = 39) of patients who completed the feedback survey (*n* = 44) reported the S-MAP intervention was completely acceptable (*n* = 22) or acceptable (*n* = 17). Three patients had no opinion and data were missing for two patients who completed the survey^d^
Fidelity of intervention delivery	Audio-recordings of a sample of patient sessions^d^	If ≤ 49% of BCTs are delivered to patients when appropriate	If 50-79% of BCTs are delivered to patients when appropriate	If ≥ 80% of BCTs are delivered to patients when appropriate	**Go:** 90.5% of BCTs were appropriately delivered to patients in the sample of audio-recorded sessions (data were available for six patients from six pharmacies)^d^
Fidelity of intervention receipt	Audio-recordings of a sample of patient sessions^d^	If ≤ 49% of delivered BCTs are received by patients as intended	If 50-79% of delivered BCTs are received by patients as intended	If ≥ 80% of delivered BCTs are received by patients as intended	**Go:** 83.2% of BCTs were received by patients as intended in the sample of audio-recorded sessions (data were available for six patients from six pharmacies)^d^
Enactment of treatment principles^d^	Audio-recordings of a sample of patient sessions^d^	If ≤ 49% of patients engaged with (or used) the delivered (or recommended) BCTs	If 50-79% of patients engaged with (or used) the delivered (or recommended) BCTs	If ≥ 80% of patients engaged with (or used) the delivered (or recommended) BCTs	**Go:** 100% of patients engaged with or used the recommended BCTs in the sample of audio-recorded sessions (data were suitable for analysis for four patients from four pharmacies)^d^

^a^This includes aspects of the study/intervention that may be modified in advance of a larger definitive trial

^b^To enable sufficient time to assess patient recruitment procedures the patient recruitment period may be extended up to a maximum of 12 months (post-training) if major ethics amendments are made during the pilot study

^c^The alternative ‘rate-related’ criterion recognises that successful patient recruitment procedures may take some time to establish

^d^The supporting data for this concept has not been reported in this paper—instead it will be reported as part of the process evaluation study paper

## Discussion

This non-randomised pilot study explored approaches to sampling, screening and recruitment of community pharmacies and older patients in two regions in the UK. It included preliminary testing of a novel web-application to guide the delivery of a tailored theory-based intervention to improve adherence to multiple medications. The feasibility of collecting data for a range of outcome measures including medication adherence, HRQOL and unplanned hospital admissions was also explored. This research will advance the existing literature by helping to address evidence gaps identified in the recent Cochrane review of adherence interventions delivered to older people prescribed multiple medicines that noted a lack of tailored interventions in this area [1].

### Sampling, screening and recruitment procedures

The procedures for identifying and recruiting pharmacies in this pilot study were successful with 12 pharmacies recruited within the allocated timeframe. One central London pharmacy withdrew as staff had insufficient time to dedicate to the study. In a future trial, it will be important to ensure that pharmacies have a realistic understanding of time required for intervention activities and adequate resources and staffing levels to participate.

Challenges were experienced with patient recruitment during the study which led to amendments to study procedures. The removal of the screening questionnaire and reduction of the eligibility age limit from 65 to 50 years increased eligibility rates from 56.8 to 76.2% and recruitment rates from 69.0 to 96.9%. Despite these improvements, the maximum target of 120 patients was not met. However, the minimum target, based on the a priori progression criteria, was met, indicating that further amendments to the recruitment protocol (e.g. additional support for patient recruitment such as using research nurses) will be required for a future cRCT. Due to the time constraints in community pharmacies, the paperwork and procedures associated with recruitment was burdensome for pharmacists, who often have limited or no experience in taking part in clinical trials. This reflects findings from other studies conducted in community pharmacies [35, 44, 45]. To enhance recruitment rates in a future trial, additional external support for pharmacists may be required, for example, support from infrastructure such as Clinical Research Network (CRN) staff [46].

Although pharmacists were advised initially to screen all older patients, a selective approach may be more pragmatic given the time restrictions in this setting. In the modified study procedures, pharmacists used a more targeted strategy and identified non-adherent patients based on their PMR dispensing data or informal discussions with patients. A review of 190 RCTs of adherence interventions, conducted in a range of settings, found that ‘the inclusion of nonadherent patients was the single feature significantly associated with effective adherence interventions’ [47]. It is therefore important that practical and cost-effective approaches to identify non-adherent patients are adopted in future research studies.

The target age in this study was reduced from 65 to 50 years based on feedback from participating pharmacists. Addressing non-adherence prior to reaching older age may have benefits that last into later life, particularly if good adherence is established in the early stages following the diagnosis of long-term conditions. UK-based ageing charities (e.g. AgeUK) offer their services to patients aged ≥ 50 years, which also supported lowering of the age limit in the study [48]. To ensure optimum adherence in the later stages of life, it may be prudent for future trials to consider targeting a wider group of patients with long-term conditions.

### Intervention delivery

The recommended minimum number of sessions was three; however, 10 patients received only one or two sessions and four patients did not attend any sessions. Most solutions were delivered during session 1 which suggests that a smaller number of sessions (e.g. two) may be sufficient to deliver this type of intervention. The intervention protocol also recommended that session 2 should take place 1-2 weeks after session 1. Based on the findings from this study, it appears that a longer time period that coincides with prescription collection frequency (e.g. 4-8 weeks) may be more appropriate.

Pharmacists identified a wide range of adherence barriers during the sessions and reported the delivery of a variety of solutions tailored to these underlying barriers. All barriers listed on the app were identified in at least one patient and no additional barriers were identified. Furthermore, all but three solutions (physical aids, review positive health goal and generic medicines leaflet) in the intervention were delivered to at least one patient. These findings highlight the relevance and comprehensiveness of the app content. This also supports the utility of the TDF and BCT mapping approach that was used to guide intervention development [11, 15]. In comparing the types of solutions delivered, a larger number of practical/social support solutions were delivered (*n* = 129) in contrast with motivation/goal-based solutions (*n* = 57); this may be explained by community pharmacists’ lack of experience in delivering motivational techniques. This reflects findings from previous research that identified a lack of training in these techniques as a potential barrier to delivering this type of adherence intervention [49]. Although training on motivational and goal-based techniques was provided during the workshops, further training and support (e.g. structured ongoing support during intervention delivery) will be required in future research studies.

### Outcome data collection

This pilot study has identified potential issues with missing data that will need to be addressed in advance of a definitive cRCT. In future research, patients who are dispensed instalment prescriptions should be excluded at baseline to help avoid this type of missing data. Given the low numbers of patients with self-reported hospitalisation data (*n* = 23) in comparison to data collected from GP records (*n* = 47), collecting data solely from GP records or administrative databases may be a better option. Some patients appeared to experience difficulties with completing the MARS-5 section of the adherence questionnaire. Strategies to enhance completion rates will need to be considered for the future (e.g. more support from researchers). There were also issues with the administration of baseline questionnaires, which was a procedure that was delegated to pharmacy staff. To minimise missing data, this process should be managed by external research support (e.g. CRN staff).

### Strengths and limitations

This study involved the systematic development and testing of a theory-based intervention, in line with UK MRC guidance for complex interventions [9], to enable community pharmacists to improve adherence to multiple medications in older adults. The use of a priori progression criteria in this study has also allowed for a transparent and impartial decision-making process on whether to proceed to a cRCT.

The study was conducted across two jurisdictions. However, it is limited by the small sample size and lack of control group, thus the presented outcome data should be interpreted with caution. No large community pharmacy chains were recruited in London (due to the time required to obtain head office approvals) and pharmacies in NI were located mainly in more deprived areas. Future research should seek to include more pharmacies to enhance representativeness of the wide diversity of pharmacy settings in the UK. The 12-month follow-up time point was removed from this study due to recruitment delays. Future research should therefore seek to include a longer follow-up period for outcome data collection.

As noted previously, three of the assessed concepts related to patient recruitment, patient retention and missing data met the ‘Amend’ progression criteria as opposed to the ‘Go’ progression criteria’. Some of the strategies discussed above will be important to consider as strategies to increase recruitment and retention rates and reduce the amount of missing data. Based on the available data, the research should progress to a cRCT in the next phase following modifications. An embedded (i.e. internal) pilot study may be required to assess changes that will need to be made to the study procedures (e.g. patient recruitment) and the intervention (e.g. app changes), as well as assessing procedures not tested in the current study such as randomisation.

## Conclusions

The results from this non-randomised pilot study support the future testing of the S-MAP intervention in community pharmacies in a definitive trial with modifications required to enhance patient recruitment, retention and data collection procedures. This study also highlights the utility of the theoretical approach that guided intervention design as it has resulted in an intervention that can identify a wide range of underlying reasons for non-adherence and lead to the delivery of tailored solutions. Future research will involve a cRCT to explore the effectiveness of the S-MAP intervention in improving adherence to multiple medications in older adults.

## Supplementary Information

**Additional file 1: Supplementary Table 1.** Region specific definitions for pharmacy types in Northern Ireland (NI) and London, England [20, 21].

**Additional file 2. **Intervention specification. **Supplementary Table 2.** Behaviour change techniques in the S-MAP intervention.

**Additional file 3. **Consort checklist. **Supplementary Table 3.** Consort checklist.

**Additional file 4. **Missing data. **Supplementary Table 4.** Missing data for primary and secondary outcome measures in the S-MAP study.

## Data Availability

The datasets used and/or analysed during the current study are available from the corresponding author on reasonable request.

## Abbreviations

BCT: Behaviour change technique
CONSORT: Consolidated Standards of Reporting Trials
CMA: Continuous medication availability
CCG: Clinical Commissioning Group
cRCT: Cluster randomised controlled trial
DPPR: Daily polypharmacy possession ratio
FTE: Full-time equivalent
GP: General practitioner
HRQOL: Health-related quality of life
HSCT: Health and Social Care Trust
IMD: Index of multiple deprivation
IQR: Interquartile range
MARS-5: Medication Adherence Report Scale
MDM: Multiple deprivation measure
MPR: Medication possession ratio
MRC: Medical Research Council
NI: Northern Ireland
NICE: National Institute for Health and Care Excellence
S-MAP: Solutions for Medication Adherence Problems
SD: Standard deviation
TDF: Theoretical domains framework
