# Supplementary material for: A non-randomised pilot study of the Solutions for Medication Adherence Problems (S-MAP) intervention in community pharmacies to support older adults adhere to multiple medications
Source: Pilot Feasibility Stud. 2021 Jan 7;7:18. doi: 10.1186/s40814-020-00762-3 (PMC7788279; doi:10.1186/s40814-020-00762-3)
Supplement: Supplementary file 1 — Additional file 1: Supplementary Table 1. Region specific definitions for pharmacy types in Northern Ireland (NI) and London, England [20, 21]. [file 40814_2020_762_MOESM1_ESM.docx]

# Additional file 1:

***Supplementary Table 1:*** *Region specific definitions for pharmacy types in Northern Ireland (NI) and London, England* [20,21]

| **Pharmacy type** | **NI definition** [21] | **England definition** [20] |
| --- | --- | --- |
| Independently owned | 1-3 pharmacies | 1-5 pharmacies |
| Small chain | 4-9 pharmacies | 6-99 pharmacies |
| Large chain | >10 pharmacies | >100 pharmacies |
