# Supplementary material for: A non-randomised pilot study of the Solutions for Medication Adherence Problems (S-MAP) intervention in community pharmacies to support older adults adhere to multiple medications
Source: Pilot Feasibility Stud. 2021 Jan 7;7:18. doi: 10.1186/s40814-020-00762-3 (PMC7788279; doi:10.1186/s40814-020-00762-3)
Supplement: Supplementary file 2 — Additional file 2. Intervention specification. Supplementary Table 2. Behaviour change techniques in the S-MAP intervention. [file 40814_2020_762_MOESM2_ESM.docx]

# Additional file 2: Intervention specification

***Supplementary Table 2:*** *Behaviour change techniques in the S-MAP intervention*

| **Behaviour change technique (BCT)** | **Specification for BCT delivery as part of the S-MAP intervention** | **Context in which the BCT be delivered? (‘core’ or ‘optional’ BCT)^a^** |
| --- | --- | --- |
| Problem-solving | The pharmacist will prompt the patient to think of factors that influence their medication-taking behaviour (e.g. being away from home) and encourage the patient to select solutions to overcome any barriers or act as facilitators of the behaviour. | All non-adherent patients (core)^a^ |
| Self-monitoring | Patients will be asked to monitor their medication use on a daily basis using a medication diary. This will include a list of the patient’s prescribed medications. |  |
| Feedback on behaviour | Based on a review of the patient’s medication diary, the pharmacist will provide feedback on the patient’s individual adherence at follow-up sessions. For example, the pharmacist might say, ‘You managed to take all of your medicines on weekdays but missed some at weekends’. |  |
| Social support (unspecified) | The pharmacist will provide, or identify others (e.g. family) who can provide, general encouragement to patients with regards to taking their medications as prescribed. |  |
| Social reward | The pharmacist will praise patients who have improved adherence and encourage continued adherence. | Patients in whom adherence has improved (optional)^a^ |
| Goal-setting (behaviour) | The pharmacist will assist patients in setting and writing down an adherence-related goal that specifies a behaviour that will be done. For example, ‘I will use my preventer inhaler every day’. | Patients deemed non-adherent at follow-up sessions (optional)^a^ |
| Action planning | A personalised plan to achieve the goal(s) set will be developed collaboratively by the patient and pharmacist. This plan can include the time, place or how often the behaviour is performed. For example, ‘When it is 9 pm and I am brushing my teeth, then I will take my simvastatin’. |  |
| Review of behaviour goal | The pharmacist and patient together will review the adherence-related goal set at the previous session and re-set or modify this. For example, if the previous goal (‘I will use my preventer inhaler every day’) was too ambitious, then the goal could be modified to one that is more achievable (‘I will use my preventer inhaler at least six days each week’). |  |
| Social support (practical)^b^ | Where necessary, additional practical support from family/friends or other healthcare professionals will be arranged. For example, family members could help the patient with organising medications into a weekly pill reminder box. | Tailored based on adherence assessment and patient need (optional)^a^ |
| Goal setting (outcome) | The pharmacist will assist patients who have low motivation in setting and writing down a goal that focuses on the positive outcomes of adherence. For example, ‘My goal is to stay out of hospital’ or ‘My goal is to have more pain free days’. |  |
| Review of outcome goal | The pharmacist and patient together will review the outcome goal that was set at the previous session. The goal will be re-set or modified. For example, it might be that the original outcome goal is not achieved by better adherence but the patient notices another unexpected benefit and decides to focus on that instead. |  |
| Information about health consequences | The pharmacist will inform patients about the benefits of taking their medications as prescribed and the risks associated with non-adherence. Patient leaflets have been designed as part of the intervention to facilitate discussions around medication concerns and generic medications and will be given to patients as part of the intervention if deemed appropriate. |  |
| Prompts and cues | The pharmacist will ask about the contexts in which forgetting is more likely and make suggestions about possible prompts. For example, a patient who routinely forgets their bedtime medications could benefit from linking medication taking to brushing their teeth. |  |
| Restructuring the physical environment | Patients may be advised to change where they store their medications or alter their home environment to facilitate adherence. For example, patients may be advised to store their night-time medications in their bedroom to facilitate adherence. |  |
| Adding objects to the environment | For patients who have difficulties with any aspect of the medication packaging or formulation/regimen and would like support, the pharmacist will provide more appropriate packaging or recommend changes to the prescriber. For example, if the patient has difficulty opening child-resistant bottle caps, then the pharmacist could supply non-child resistant bottle caps. |  |
| Instruction on how to perform the behaviour | Patients may be advised on strategies to improve the process of taking medications (e.g. such as tips to aid swallowing medications where difficulties are noted) |  |

*^a^‘Core’ BCTs were recommended for delivery to all non-adherent patients in the study. ‘Optional’ BCTs were delivered based on each individual patient’s needs including the underlying reasons for non-adherence and improvements in adherence scores*
