# Supplementary material for: A non-randomised pilot study of the Solutions for Medication Adherence Problems (S-MAP) intervention in community pharmacies to support older adults adhere to multiple medications
Source: Pilot Feasibility Stud. 2021 Jan 7;7:18. doi: 10.1186/s40814-020-00762-3 (PMC7788279; doi:10.1186/s40814-020-00762-3)
Supplement: Supplementary file 4 — Additional file 4. Missing data. Supplementary Table 4. Missing data for primary and secondary outcome measures in the S-MAP study. [file 40814_2020_762_MOESM4_ESM.docx]

# Additional file 4: Missing data

***Supplementary Table 4:*** *Missing data for primary and secondary outcome measures in the S-MAP study*

| **Data source** | **N of patients with missing data/excluded data** | **% missing** |
| --- | --- | --- |
| **Primary outcome** | | |
| **Dispensing records** *(6 months pre and 6 months post session 1)* | 16 | 26.7% |
| **Lu item** | | |
| - Baseline | 12 | 20.0% |
| - Follow-up | 17 | 28.3% |
| **MARS-5-baseline** | | |
| - Item 1 | 12 | 20.0% |
| - Item 2 | 13 | 21.7% |
| - Item 3 | 13 | 21.7% |
| - Item 4 | 13 | 21.7% |
| - Item 5 | 14 | 23.3% |
| **MARS-5-follow-up** | | |
| - Item 1 | 25 | 41.7% |
| - Item 2 | 29 | 48.3% |
| - Item 3 | 29 | 48.3% |
| - Item 4 | 25 | 41.7% |
| - Item 5 | 29 | 48.3% |
| **Secondary outcomes** | | |
| **EQ-5D-5L- baseline** | | |
| - Mobility | 17 | 28.3% |
| - Self-care | 16 | 26.7% |
| - Usual activities | 17 | 28.3% |
| - Pain/discomfort | 16 | 26.7% |
| - Anxiety/depression | 16 | 26.7% |
| - EQ-VAS | 15 | 25.0% |
| **EQ-5D-5L- follow-up** | | |
| - Mobility | 17 | 28.3% |
| - Self-care | 17 | 28.3% |
| - Usual activities | 19 | 31.7% |
| - Pain/discomfort | 17 | 28.3% |
| - Anxiety/depression | 17 | 28.3% |
| - EQ-VAS | 17 | 28.3% |
| **Patient-reported unplanned hospital admissions** | | |
| - Baseline | 24 | 40.0% |
| - Follow-up | 21 | 35.0% |
| **GP-reported unplanned hospital admissions** *(6 months pre and 6 months post session 1)* | 13 | 21.7% |
| **Overall** | - | 30.1% |
